# Supplementary material for: Durability of the benefit of vagus nerve stimulation in markedly treatment-resistant major depression: a RECOVER trial report
Source: Int J Neuropsychopharmacol. 2026 Jan 13;29(1):pyaf080. doi: 10.1093/ijnp/pyaf080 (PMC12799215; doi:10.1093/ijnp/pyaf080)
Supplement: Resubmission_RECOVER_Durability_Manuscript_7a_Supplemental_120825_pyaf080 [file resubmission_recover_durability_manuscript_7a_supplemental_120825_pyaf080.pdf]

Supplemental Materials

Table of Contents

Title 2
Authors 2
Supplemental Materials 4
Supplemental Methods 1 14
Supplemental Methods 2 18

Supplemental Materials

Durability of the Benefit of Vagus Nerve Stimulation in Markedly Treatment-Resistant Major Depression: A RECOVER Trial Report

Charles R. Conway, MD[1], A. John Rush, MD[2,3], Scott T. Aaronson, MD[4], Mark T. Bunker, PharmD[5], Charles Gordon, MS[5], Mark S. George, MD[6,7], Patricio Riva-Posse, MD[8], Rebecca M. Allen, MD[9], Ziad Nahas, MD[10], Christopher L. Kriedt, BSN, BS[1], John Zajecka, MD[11,12], David L. Dunner, MD[13], João Quevedo, MD, PhD[14], Yvette Sheline, MD[15], Walter Duffy, MD[16], Brian J. Mickey, MD, PhD[17], Mary Stedman, MD[18], Gustavo Alva, MD[19], Lucian Manu, MD[20], Quyen Tran, BS, MBA[5], Charles F. Zorumski, MD[1], Matthew Macaluso, DO[21], Michael Banov, MD[22], Cristina Cusin, MD[23], Jeffrey I. Bennett, MD[24], Hunter Brown, LMSW[1], Jeffrey Way, BS[5], Olivia Shy, MS[5], Ying-Chieh (Lisa) Lee, MS[5], R. Hamish McAllister-Williams, MD, PhD[25-27], Roger S. McIntyre, MD [28], Harold A. Sackeim, PhD[6]

1 Department of Psychiatry, Washington University in St Louis, St Louis, MO, USA

2 Duke-NUS Medical School, Singapore

3 Curbstone Consultant LLC, Dallas, TX, USA

4 Institute for Advanced Diagnostics and Therapeutics, Sheppard Pratt Health System, Baltimore, MD, USA

5 LivaNova PLC (or a subsidiary), London, UK

6 Department of Psychiatry, Medical University of South Carolina, Charleston, SC, USA

7 Ralph H. Johnson VA Health Care System, Charleston, SC, USA

8 Department of Psychiatry and Behavioral Sciences, Emory University School of Medicine, Atlanta, GA, USA

9 Seattle Neuropsychiatric Treatment Center, Seattle, WA, USA

10 University of Minnesota, Minneapolis, MN, USA

[11] Department of Psychiatry and Behavioral Sciences, Rush University Medical Center, Chicago, IL, USA

[12] Psychiatric Medicine Associates, LLC, Skokie, IL, USA

[13] Center for Anxiety and Depression, Mercer Island, WA, USA

[14] Center for Interventional Psychiatry, Faillace Department of Psychiatry and Behavioral Sciences, McGovern Medical School, The University of Texas Health Science Center at Houston (UTHealth Houston), Houston, TX, USA

[15] University of Pennsylvania Perelman School of Medicine, Philadelphia, PA, USA

[16] Alivation Research, Lincoln, NE, USA

[17] Department of Psychiatry, Huntsman Mental Health Institute, University of Utah, Salt Lake City, UT, USA

[18] Stedman Clinical Trials, Tampa, FL, USA

[19] ATP Clinical Research, Costa Mesa, CA, USA

[20] Department of Psychiatry and Behavioral Health, Renaissance School of Medicine at Stony Brook University, Stony Brook, NY, USA

[21] University of Alabama, Birmingham, AL, USA

[22] PsychAtlanta Research Center, Marietta, GA, USA

[23] Mass General Psychiatry: Depression Clinical & Research Program, Boston, MA, USA

[24] SIU Neuroscience Institute, Springfield, IL, USA

[25] Translational and Clinical Research Institute, Newcastle University, Newcastle upon Tyne, UK

[26] Northern Centre for Mood Disorders, Newcastle University, Newcastle upon Tyne, UK

[27] Cumbria, Northumberland, Tyne and Wear NHS Foundation Trust, Newcastle upon Tyne, UK

[28] Department of Psychiatry, University of Toronto, Toronto, ON, Canada

Table S1. Rates of each clinical outcome at 12, 18, and 24 months across the sample

Outcome Classification	12 Months	18 Months	24 Months
		**MADRS**	
N	213	208	186
≥MB	45.5% (97)	50.5% (105)	53.2% (99)
SB	29.1% (62)	32.2% (67)	33.3% (62)
MB	16.4% (35)	18.3% (38)	19.9% (37)
Remission	16.4% (35)	17.3% (36)	21.0% (39)
NB	54.5% (116)	49.5% (103)	46.8% (87)
		**QIDS-C**	
N	213	208	186
≥MB	57.3% (122)	58.2% (121)	61.8% (115)
SB	35.2% (75)	36.5% (76)	40.9% (76)
MB	22.1% (47)	21.6% (45)	21.0% (39)
Remission	19.7% (42)	22.1% (46)	24.2% (45)
NB	42.7% (91)	41.8% (87)	38.2% (71)
		**QIDS-SR**	
N	213	208	186
≥MB	60.6% (129)	57.2% (119)	58.6% (109)
SB	38.5% (82)	38.9% (81)	40.9% (76)
MB	22.1% (47)	18.3% (38)	17.7% (33)
Remission	24.9% (53)	20.7% (43)	23.1% (43)
NB	39.4% (84)	42.8% (89)	41.4% (77)
		**CGI-I**	
N	214	209	190
≥MB	68.7% (147)	78.5% (164)	77.4% (147)
SB	40.2% (86)	47.4% (99)	51.6% (98)
MB	28.5% (61)	31.1% (65)	25.8% (49)
Remission	16.8% (36)	15.8% (33)	22.1% (42)
NB	31.3% (67)	21.5% (45)	22.6% (43)
		**Function (WPAI Item 6)**	
N	208	203	180
MB	57.2% (119)	65.0% (132)	60.0% (108)
NB	42.8% (89)	35.0% (71)	40.0% (72)
		**QoL (Mini-Q-LES-Q)**	
N	206	200	178
MB	63.1% (130)	67.0% (134)	66.3% (118)
NB	36.9% (76)	33.0% (66)	33.7% (60)
		**Tripartite Metric**	
N	200	194	171
≥MB	80.0% (160)	82.0% (159)	81.9% (140)

SB	61.0% (122)	64.9% (126)	65.5% (112)
MB	19.0% (38)	17.6% (33)	19.7% (28)
NB	20.0% (40)	18.0% (35)	18.1% (31)

Data presented as % (n).

Abbreviations: CGI-I, Clinical Global Impression–Improvement; MADRS, Montgomery-Åsberg Depression Rating Scale; MB, meaningful benefit; Mini-Q-LES-Q, 7-item subset of the Quality of Life Enjoyment and Satisfaction Questionnaire; NB, no meaningful benefit; QIDS-C, Quick Inventory of Depressive Symptomatology–Clinician; QIDS-SR, Quick Inventory of Depressive Symptomatology–Self-Report; SB, substantial benefit; WPAI, Work Productivity and Activity Impairment Questionnaire.

Table S2. Proportion of participants with missing data at month 24 based on month 12 outcome

	Participants at 12 Months			Missing Data at 24 Months	
**Outcome Measure**	**N**	**≥MB**	**NB**	**≥MB at 12 Months**	**NB at 12 Months**
**MADRS**	221	97	124	15.5%	16.1%
**QIDS-C**	221	124	97	16.1%	15.5%
**QIDS-SR**	220	130	90	13.1%	18.9%
**CGI-I**	221	149	72	13.4%	15.3%
**Function (WPAI item 6)**	217	121	96	16.5%	17.7%
**QoL (Mini-Q-LES-Q)**	212	131	81	14.5%	18.5%
**Tripartite metric**	209	164	45	17.7%	20.0%

Abbreviations: CGI-I, Clinical Global Impression–Improvement; MADRS, Montgomery-Åsberg Depression Rating Scale; MB, meaningful benefit; Mini-Q-LES-Q, 7-item subset of the Quality of Life Enjoyment and Satisfaction Questionnaire; NB, no meaningful benefit; QIDS-C, Quick Inventory of Depressive Symptomatology–Clinician; QIDS-SR, Quick Inventory of Depressive Symptomatology–Self-Report; QoL, quality of life; WPAI, Work Productivity and Activity Impairment Questionnaire.

Table S3. Percentage of participants who maintained meaningful benefit or substantial benefit at 18- and 24-month follow-up

Maintenance of ≥MB in Participants With ≥MB at 12 Months				
**Outcome Measure (N at Month 12)**	**≥MB at 18 Months**		**≥MB at 24 Months**	
	**Observed**	**Imputed**	**Observed**	**Imputed**
MADRS (97)	83.0% (78/94)	80.4%	82.9% (68/82)	70.1%
QIDS-C (122)	81.5% (97/119)	79.5%	80.8% (84/104)	68.9%
QIDS-SR (129)	78.0% (99/127)	76.7%	79.6% (90/113)	69.8%
CGI-I (147)	89.0% (129/145)	87.8%	85.3% (110/129)	74.8%
QoL (Mini-Q-LES-Q; 130)	87.5% (112/128)	86.2%	81.3% (91/112)	70.0%
Function (WPAI item 6; 119)	83.1% (98/118)	82.4%	79.2% (80/101)	67.2%
Tripartite metric (160)	89.2% (140/157)	87.5%	89.6% (121/135)	75.6%
**Maintenance of ≥MB in Participants With SB at 12 Months**				
**Outcome Measure (N at Month 12)**	**≥MB at 18 Months**		**≥MB at 24 Months**	
	**Observed**	**Imputed**	**Observed**	**Imputed**
MADRS (62)	95.0% (57/60)	91.9%	92.6% (50/54)	80.6%
QIDS-C (75)	87.8% (65/74)	86.7%	90.3% (56/62)	74.7%
QIDS-SR (82)	92.6% (74/80)	90.2%	87.3% (62/71)	75.6%
CGI-I (86)	95.3% (81/85)	94.2%	92.2% (71/77)	82.6%
Tripartite metric (122)	93.3% (111/119)	91.0%	94.2% (97/103)	79.5%
**Maintenance of SB in Participants With SB at 12 Months**				
**Outcome Measure (N at Month 12)**	**SB at 18 Months**		**SB at 24 Months**	
	**Observed**	**Imputed**	**Observed**	**Imputed**
MADRS (62)	75.0% (45/60)	72.6%	74.1% (40/54)	64.5%
QIDS-C (75)	74.3% (55/74)	73.3%	79.0% (49/62)	65.3%
QIDS-SR (82)	78.8% (63/80)	76.8%	74.6% (53/71)	64.6%
CGI-I (86)	80.0% (68/85)	79.1%	83.1% (64/77)	74.4%
Tripartite metric (122)	84.9% (101/119)	82.8%	80.6% (83/103)	68.0%

Observed: Proportions were calculated using all available data at each visit.
Imputed: Proportions were calculated using the number of participants with available data at the month 12 visit as the denominator; missing data were considered as not meeting the response outcome.

Abbreviations: CGI-I, Clinical Global Impression–Improvement; MADRS, Montgomery-Åsberg Depression Rating Scale; MB, meaningful benefit; Mini-Q-LES-Q, 7-item subset of the Quality of Life Enjoyment and Satisfaction Questionnaire; QIDS-C, Quick Inventory of Depressive Symptomatology–Clinician; QIDS-SR, Quick Inventory of Depressive Symptomatology–Self-Report; QoL, quality of life; SB, substantial benefit; WPAI, Work Productivity and Activity Impairment Questionnaire.

Table S4. Mean (SD) concomitant psychotropic medications at month 12 and last follow-up (month 18 or 24) for the total sample, participants with meaningful benefit on the tripartite metric (score ≥1), and participants with substantial benefit on the CGI-I (score ≤2)

	**Total Sample (N=214)**		
	**Month 12**	**Last Follow-Up[b]**	***P***
Total Psychotropics[a]	4.6 (2.4)	4.6 (2.5)	0.892
Antidepressants	1.8 (0.9)	1.8 (0.9)	1.000
Anxiolytics	0.8 (0.8)	0.8 (0.8)	0.224
Antipsychotics	0.5 (0.6)	0.5 (0.6)	0.533
Analgesics	0.4 (0.8)	0.4 (0.9)	0.096
Anticonvulsants	0.5 (0.7)	0.5 (0.7)	0.819
Hypnotics	0.2 (0.5)	0.2 (0.5)	0.083
Stimulants	0.3 (0.6)	0.4 (0.6)	0.286
	**MB on the Tripartite Metric (n=142)**		
	**Month 12**	**Last Follow-Up[b]**	***P***
Total Psychotropics[a]	4.6 (2.5)	4.6 (2.6)	0.750
Antidepressants	1.8 (0.9)	1.8 (0.9)	1.000
Anxiolytics	0.8 (0.8)	0.8 (0.8)	0.452
Antipsychotics	0.4 (0.6)	0.4 (0.6)	0.740
Analgesics	0.4 (0.9)	0.5 (1.0)	0.158
Anticonvulsants	0.5 (0.7)	0.5 (0.7)	1.000
Hypnotics	0.2 (0.5)	0.2 (0.5)	0.083
Stimulants	0.3 (0.6)	0.4 (0.6)	0.250
	**SB on the CGI-I (n=70)**		
	**Month 12**	**Last Follow-Up[b]**	***P***
Total Psychotropics[a]	4.6 (2.8)	4.6 (3.0)	0.816
Antidepressants	1.9 (0.8)	1.9 (0.8)	0.621
Anxiolytics	0.8 (0.9)	0.8 (0.8)	0.798
Antipsychotics	0.4 (0.6)	0.4 (0.6)	0.567
Analgesics	0.4 (0.9)	0.4 (1.0)	0.182
Anticonvulsants	0.4 (0.8)	0.5 (0.8)	0.321
Hypnotics	0.2 (0.5)	0.2 (0.5)	0.083
Stimulants	0.2 (0.5)	0.2 (0.5)	0.567

[a]Includes all categories noted above.
[b]Last follow-up is at month 24, if available, otherwise it is at month 18.

Abbreviations: CGI-I, Clinical Global Impression–Improvement; MB, meaningful benefit; SB, substantial benefit; SD, standard deviation.

Table S5. Proportion of participants receiving interventional treatment at any time **during the 90 days** prior to month 12 and last follow-up (month 18 or 24) for the total sample, participants with meaningful benefit on the tripartite metric (score ≥1), and participants with substantial benefit on the CGI-I (score ≤2)

	**Total Sample (N=214)**	
	**Month 12**	**Last Follow-Up[a]**
**ECT**	6 (2.8%)	6 (2.8%) [1]
**TMS**	2 (0.9%)	3 (1.4%) [1]
**Ketamine/esketamine**	23 (10.7%)	24 (11.2%) [2]
	**MB on the Tripartite Metric (n=142)**	
	**Month 12**	**Last Follow-Up[a]**
**ECT**	2 (1.4%)	1 (0.7%) [0]
**TMS**	1 (0.7%)	1 (0.7%) [0]
**Ketamine/esketamine**	14 (9.9%)	15 (10.6%) [2]
	**SB on the CGI-I (n=70)**	
	**Month 12**	**Last Follow-Up[a]**
**ECT**	3 (4.3%)	2 (2.9%) [0]
**TMS**	2 (2.9%)	2 (2.9%) [0]
**Ketamine/esketamine**	6 (8.6%)	7 (10.0%) [1]

Counts in [ ] brackets indicate participants using interventional treatment at the visit date who were not counted at month 12.

[a]Last follow-up is at month 24, if available, otherwise it is at month 18.

Abbreviations: CGI-I, Clinical Global Impression–Improvement; ECT, electroconvulsive therapy; MB, meaningful benefit; SB, substantial benefit; TMS, transcranial magnetic stimulation.

Figure S1. Alluvial diagram of depressive symptom measures, including (A) MADRS, (B) QIDS-C, and (C) QIDS-SR findings over 18 and 24 months

A. MADRS

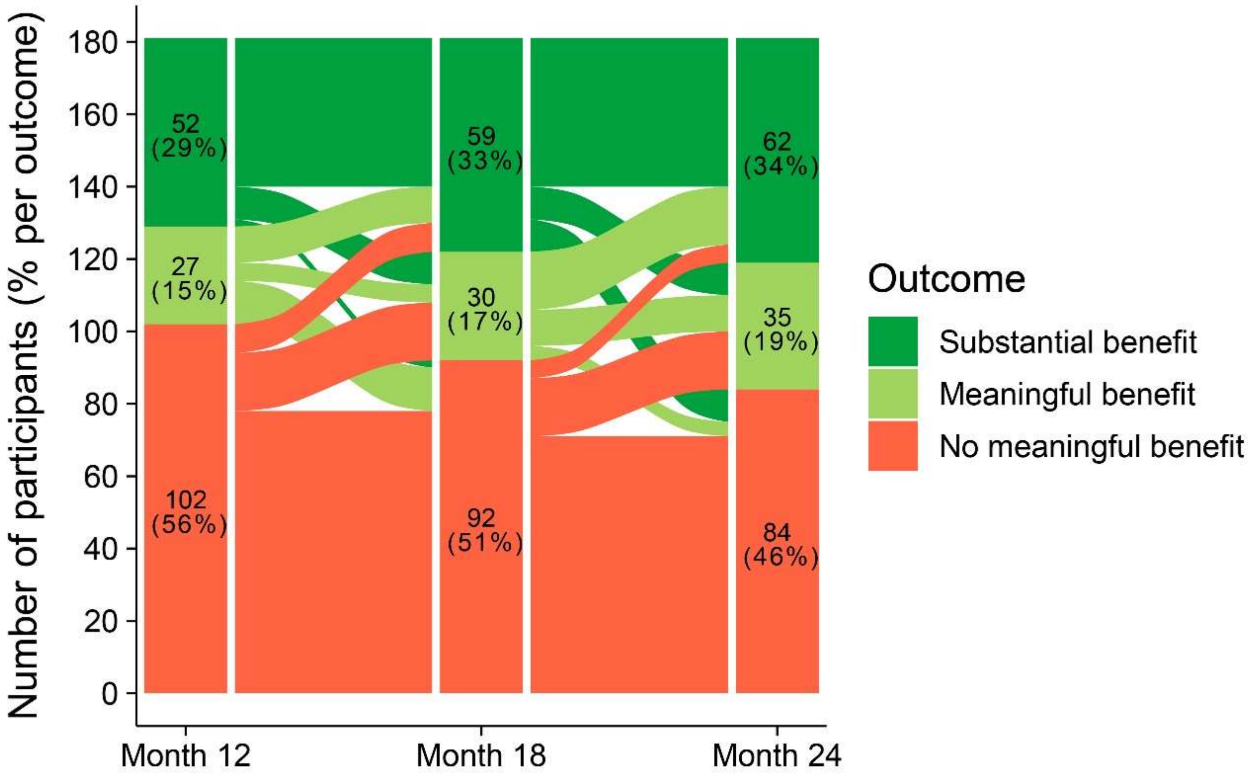

B. QIDS-C

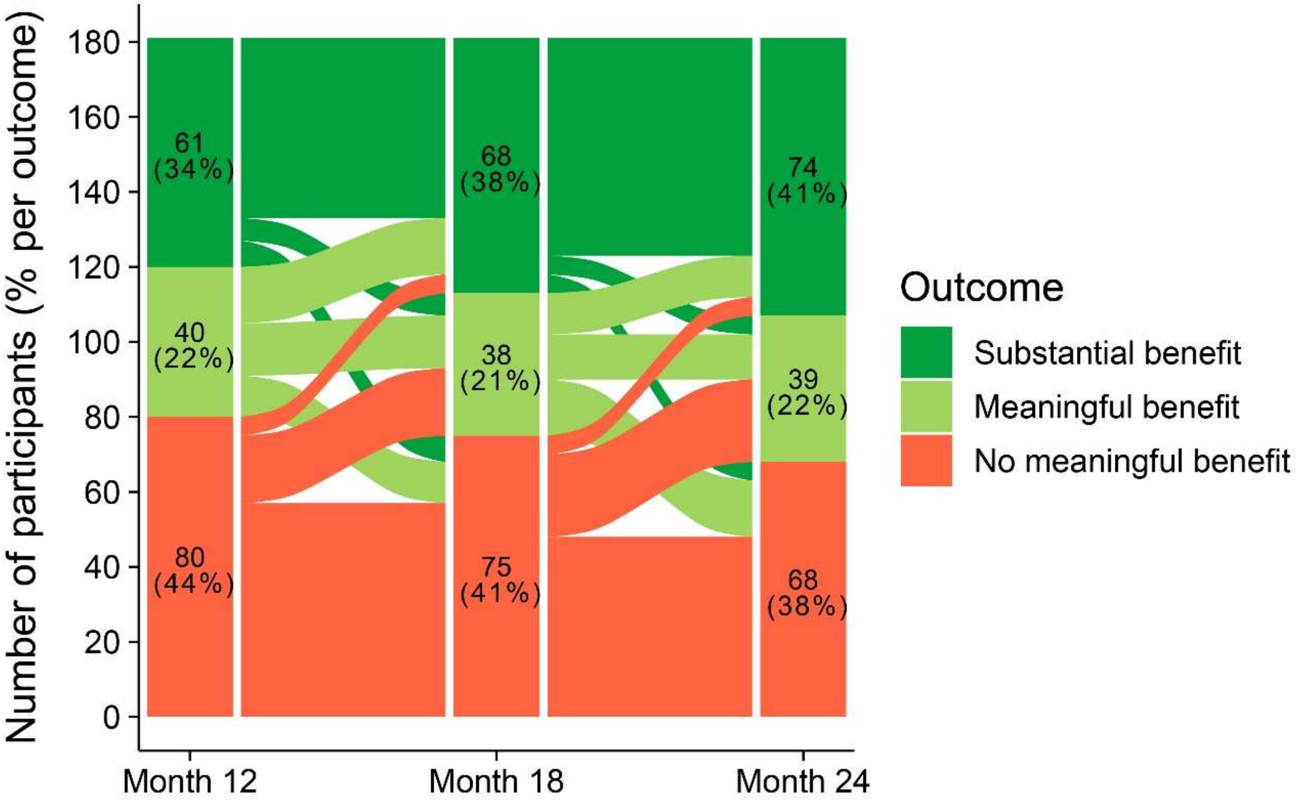
Number of participants (% per outcome)
180
160
140
120
100
80
60
40
20
0
61 (34%)
40 (22%)
80 (44%)
68 (38%)
38 (21%)
75 (41%)
74 (41%)
39 (22%)
68 (38%)
Month 12
Month 18
Month 24
Outcome
Substantial benefit
Meaningful benefit
No meaningful benefit

C. QIDS-SR

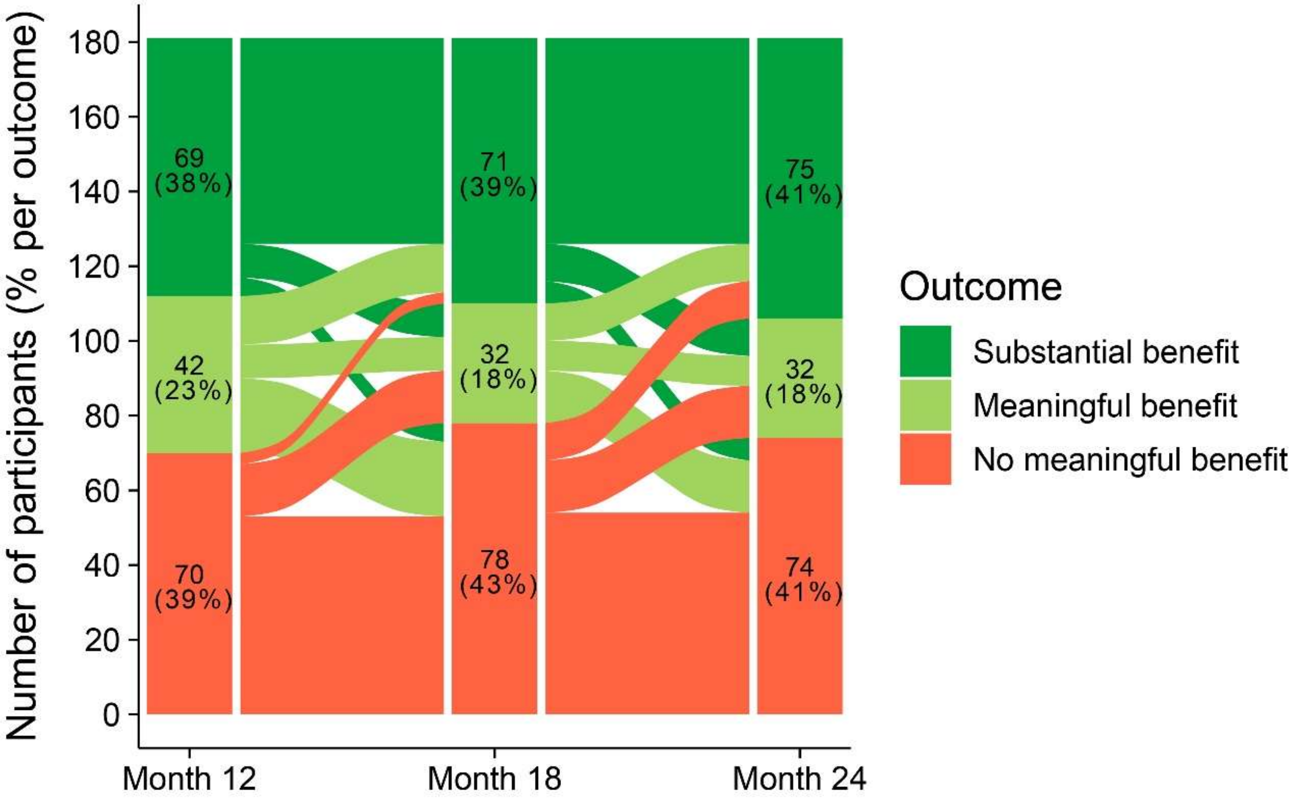

Abbreviations: MADRS, Montgomery-Åsberg Depression Rating Scale; QIDS-C, Quick Inventory of Depressive Symptomatology–Clinician; QIDS-SR, Quick Inventory of Depressive Symptomatology–Self-Report.

Figure S2. Alluvial diagram of (A) Mini-Q-LES-Q and (B) WPAI item 6 findings over 18 and 24 months

A. Mini-Q-LES-Q

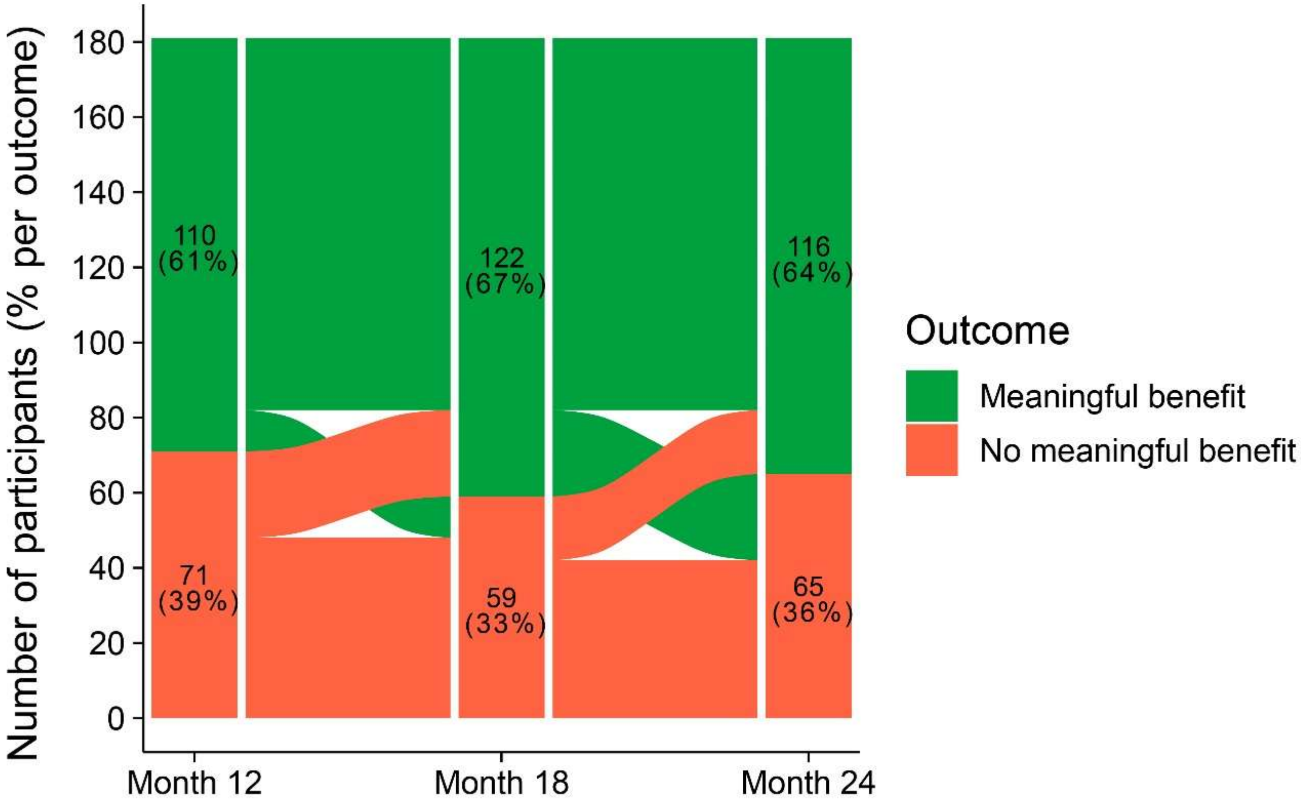

B. WPAI item 6

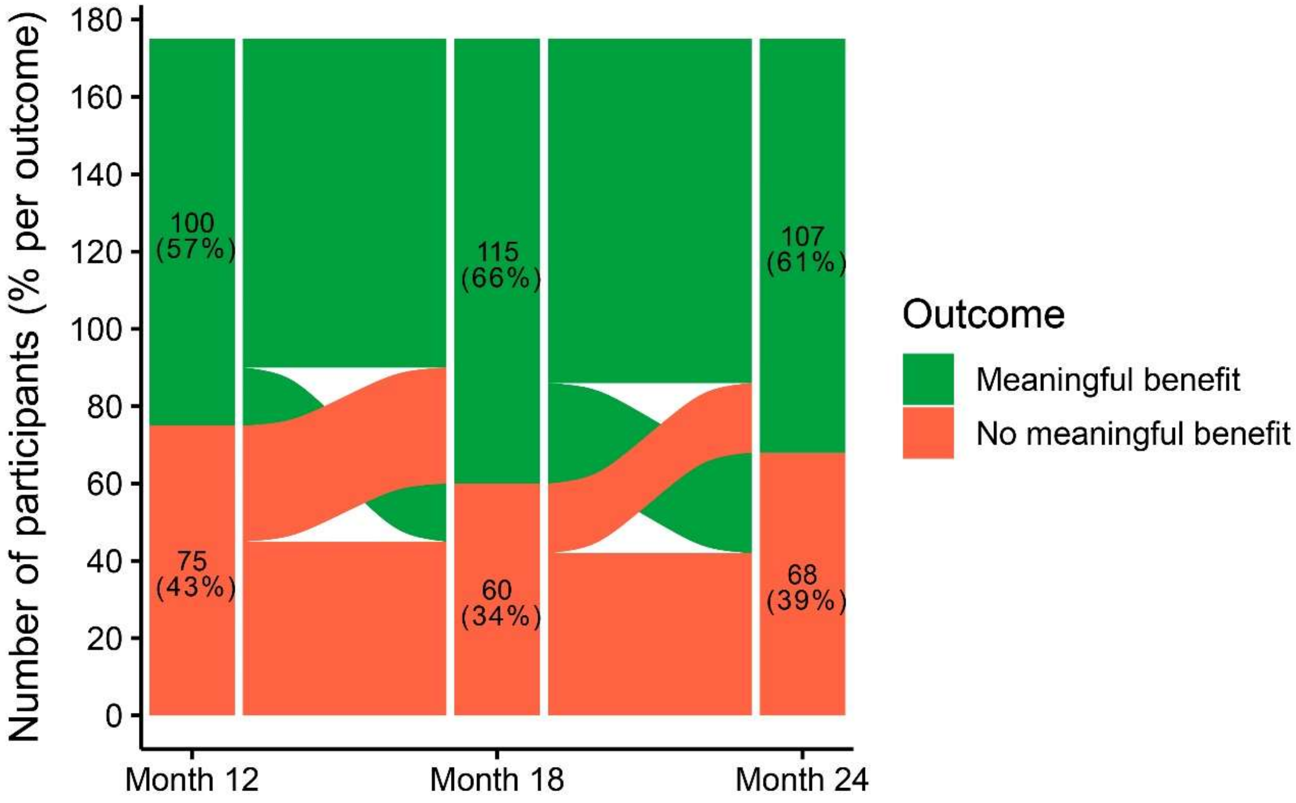

Abbreviations: Mini-Q-LES-Q, 7-item subset of the Quality of Life Enjoyment and Satisfaction Questionnaire; WPAI, Work Productivity and Activity Impairment Questionnaire.

Supplemental Methods 1. Identification and classification of concomitant psychotropic medications and interventional treatments in the RECOVER trial

Concomitant Medications. All ongoing medications were documented at each assessment occasion based on patient reporting and categorized into pharmacological classes with modification of the WHO Anatomical Therapeutic Chemical (ATC) classification system. Information on the dosage of medications was not examined in the current study. The universe of medications considered was restricted to ATC Level 1 N: Nervous system, that is, compounds with psychotropic properties. Modifications to the WHO ATC classification system reflected the consensus judgement of the 6 co-authors with expertise in clinical pharmacology, neuromodulation, and the treatment of treatment-resistant depression (TRD; SA, MB, CC, HMW, HAS, and AJR). The RECOVER database included 209 entries of medications or medication combinations categorized by the WHO ATC classification system as nervous system agents. Each entry was reviewed, and 14 that concerned agents with questionable psychotropic properties or relevance to therapeutic effects in TRD were identified. These excluded medications are listed below.

Medications not considered psychotropics

**Medication**	**Indication or Use**
BETHANECHOL CHLORIDE	Postoperative and postpartum urinary retention
BUPIVACAINE	Anesthetic
CANNABIDIOL/ CANNABIS SATIVA	Treat seizures associated with Lennox-Gastaut syndrome, Dravet syndrome, or tuberous sclerosis complex
CEVIMELINE	Treatment for dry mouth

DIPHENHYDRAMINE/ DIPHENHYDRAMINE HYDROCHLORIDE	Antihistamine
EPINEPHRINE	For emergency treatment of allergic reactions, including anaphylaxis
GAMMA-AMINOBUTYRIC ACID; MAGNESIUM OXIDE; MATRICARIA CHAMOMILLA; MELATONIN; PASSIFLORA INCARNATA; TRYPTOPHAN, L-; VALERIANA OFFICINALIS ROOT EXTRACT; WITHANIA SOMNIFERA EXTRACT	Supplement for relaxation
LIDOCAINE	Local anesthetic
METOPROLOL	Treatment for hypertension
PILOCARPINE	Treatment for dry mouth
PYRIDOSTIGMINE	Treatment for myasthenia gravis
THIOCTIC ACID	Treatment for peripheral diabetic polyneuropathy
TIZANIDINE	Management of spasticity
WITHANIA SOMNIFERA	Supplement for stress and anxiety, sleep, male infertility, and athletic performance

The WHO ATC classification system further categorizes medications by chemical, pharmacological, or therapeutic subgroup (eg, analgesics, antidepressants). However, the same medication may receive multiple classifications based on differing intended uses. For example, in the RECOVER database, there were 3 entries for amitriptyline, which was classified as an analgesic, antidepressant, and anxiolytic. The statistical analyses required that each medication be assigned to 1 class so that additions and subtractions can be counted only once per medication. Therefore, consensus was also achieved on the class assigned to each medication. These classifications reflected widely accepted pharmacological distinctions. For example, amitriptyline was coded as an antidepressant, and valproic acid as an anticonvulsant

(antiepileptic). Since the WHO ACT classification system does not contain a category for mood stabilizers, lithium was placed in the antidepressant class in place of its WHO assignment as an antipsychotic. The WHO class of anesthetics was removed, as 2 of 3 entries pertained to local anesthetics and the third to ketamine, which was reclassified in the antidepressant grouping.

The medication classes derived from the WHO ACT classification system are listed below. Due to their infrequency of use and limited relevance for therapeutic effects in TRD, the classes of antidementia drugs, anti-Parkinson drugs, and other nervous system drugs (for addictive disorders or miscellaneous) were combined into a single class, termed "other." The total number of psychotropic medications at an assessment occasion corresponded to the sum of number of medications in the classes of analgesics, anticonvulsants, antidepressants, antipsychotics, anxiolytics, hypnotics, stimulants, and other.

WHO medication classes and corresponding RECOVER classes

WHO ACT Class	RECOVER Class
Analgesics	Analgesics
Antidementia drugs	Other
Anti-Parkinson drugs	Other
Antidepressants	Antidepressants
Antiepileptics	Anticonvulsants
Antipsychotics	Antipsychotics
Anxiolytics	Anxiolytics
Hypnotics and sedatives	Hypnotics
Other nervous system drugs used in addictive disorders	Other
Other nervous system drugs miscellaneous	Other
Psychostimulants, agents used for ADHD, and nootropics	Stimulants

The complete list of psychotropic medication entries in RECOVER and their classifications are presented in Supplemental Methods 2.

Concomitant Interventional Psychiatry Treatments

At each assessment occasion, ongoing treatment with esketamine or ketamine, electroconvulsive therapy (ECT), or transcranial magnetic stimulation (TMS) was also documented. There is substantial evidence regarding the efficacy of these interventions, specifically in treatment of TRD. Previous research demonstrated in the RECOVER sample that a history of treatment with ECT or TMS was prognostic of outcomes after the first year. Thus, determination of whether the use of interventional psychiatry treatments changed over the second year of RECOVER is consequential in interpreting the durability findings.

After completion of the first year's blinded, randomized phase, there was no restriction on concomitant treatments. These interventional treatments could be administered as rescue interventions, continuation/maintenance treatment, or acute courses. This study identified the number of participants receiving each type of intervention (ketamine, ECT, and/or TMS) at the month-12 and last follow-up time points, and the number of patients who added or subtracted these interventions at end point relative to month 12.

Supplemental Methods 2. The complete list of psychotropic medication entries in RECOVER and their classifications

Standardized Medication Name	WHO Drug ATC Level 1	WHO Drug ATC Level 2	WHO Drug ATC Level 3	**Classification in RECOVER**
ACETAZOLAMIDE	NERVOUS SYSTEM	ANTIEPILEPTICS	ANTIEPILEPTICS	ANTIEPILEPTICS
ACETYLSALICYLIC ACID	NERVOUS SYSTEM	ANALGESICS	OTHER ANALGESICS AND ANTIPYRETICS	ANALGESICS
ACETYLSALICYLIC ACID;CAFFEINE;PARACETAMOL	NERVOUS SYSTEM	ANALGESICS	OTHER ANALGESICS AND ANTIPYRETICS	ANALGESICS
ACETYLSALICYLIC ACID;CHLORPHENAMINE;PHENYLEPHRINE	NERVOUS SYSTEM	ANALGESICS	OTHER ANALGESICS AND ANTIPYRETICS	ANALGESICS
AGOMELATINE	NERVOUS SYSTEM	PSYCHOANALEPTICS	ANTIDEPRESSANTS	ANTIDEPRESSANTS
ALPRAZOLAM	NERVOUS SYSTEM	PSYCHOLEPTICS	ANXIOLYTICS	ANXIOLYTICS
AMANTADINE	NERVOUS SYSTEM	ANTI-PARKINSON DRUGS	DOPAMINERGIC AGENTS	OTHER
AMITRIPTYLINE	NERVOUS SYSTEM	PSYCHOANALEPTICS	ANTIDEPRESSANTS	ANTIDEPRESSANTS
AMOXAPINE	NERVOUS SYSTEM	PSYCHOANALEPTICS	ANTIDEPRESSANTS	ANTIDEPRESSANTS
AMPHETAMINE	NERVOUS SYSTEM	PSYCHOANALEPTICS	PSYCHOSTIMULANTS, AGENTS USED FOR ADHD AND NOOTROPICS	STIMULANTS
AMPHETAMINE;DEXAMPHETAMINE	NERVOUS SYSTEM	PSYCHOANALEPTICS	PSYCHOSTIMULANTS, AGENTS USED FOR ADHD AND NOOTROPICS	STIMULANTS
ARIPIPRAZOLE	NERVOUS SYSTEM	PSYCHOLEPTICS	ANTIPSYCHOTICS	ANTIPSYCHOTICS
ARMODAFINIL	NERVOUS SYSTEM	PSYCHOANALEPTICS	PSYCHOSTIMULANTS, AGENTS USED FOR ADHD AND NOOTROPICS	STIMULANTS
ASENAPINE	NERVOUS SYSTEM	PSYCHOLEPTICS	ANTIPSYCHOTICS	ANTIPSYCHOTICS
ATOGEPANT	NERVOUS SYSTEM	ANALGESICS	ANTIMIGRAINE PREPARATIONS	ANALGESICS
ATOMOXETINE	NERVOUS SYSTEM	PSYCHOANALEPTICS	PSYCHOSTIMULANTS, AGENTS USED FOR ADHD AND NOOTROPICS	STIMULANTS
BACLOFEN;GABAPENTIN;LIDOCAINE	NERVOUS SYSTEM	ANALGESICS	OTHER ANALGESICS AND ANTIPYRETICS	ANALGESICS
BENZATROPINE	NERVOUS SYSTEM	ANTI-PARKINSON DRUGS	ANTICHOLINERGIC AGENTS	OTHER
BOTULINUM TOXIN TYPE A	NERVOUS SYSTEM	ANALGESICS	ANTIMIGRAINE PREPARATIONS	ANALGESICS
BREXPIPRAZOLE	NERVOUS SYSTEM	PSYCHOLEPTICS	ANTIPSYCHOTICS	ANTIPSYCHOTICS
BUPRENORPHINE	NERVOUS SYSTEM	ANALGESICS	DRUGS USED IN ADDICTIVE DISORDERS	OTHER
BUPRENORPHINE;NALOXONE	NERVOUS SYSTEM	OTHER NERVOUS SYSTEM DRUGS	DRUGS USED IN ADDICTIVE DISORDERS	OTHER
BUPROPION	NERVOUS SYSTEM	PSYCHOANALEPTICS	ANTIDEPRESSANTS	ANTIDEPRESSANTS
BUPROPION;DEXTROMETHORPHAN	NERVOUS SYSTEM	PSYCHOANALEPTICS	ANTIDEPRESSANTS	ANTIDEPRESSANTS
BUSPIRONE	NERVOUS SYSTEM	PSYCHOLEPTICS	ANXIOLYTICS	ANXIOLYTICS
BUTALBITAL;CAFFEINE;PARACETAMOL	NERVOUS SYSTEM	ANALGESICS	OTHER ANALGESICS AND ANTIPYRETICS	ANALGESICS
BUTALBITAL;PARACETAMOL	NERVOUS SYSTEM	ANALGESICS	OTHER ANALGESICS AND ANTIPYRETICS	ANALGESICS

CAFFEINE;CODEINE;PARACETAMOL	NERVOUS SYSTEM	ANALGESICS	OPIOIDS	ANALGESICS
CARBAMAZEPINE	NERVOUS SYSTEM	PSYCHOLEPTICS	ANTIPSYCHOTICS	ANTIEPILEPTICS
CARBIDOPA MONOHYDRATE;LEVODOPA	NERVOUS SYSTEM	ANTI-PARKINSON DRUGS	DOPAMINERGIC AGENTS	OTHER
CARIPRAZINE	NERVOUS SYSTEM	PSYCHOLEPTICS	ANTIPSYCHOTICS	ANTIPSYCHOTICS
CHLORDIAZEPOXIDE	NERVOUS SYSTEM	PSYCHOLEPTICS	ANXIOLYTICS	ANXIOLYTICS
CHLORPROMAZINE	NERVOUS SYSTEM	PSYCHOLEPTICS	ANTIPSYCHOTICS	ANTIPSYCHOTICS
CITALOPRAM	NERVOUS SYSTEM	PSYCHOANALEPTICS	ANTIDEPRESSANTS	ANTIDEPRESSANTS
CLOMIPRAMINE	NERVOUS SYSTEM	PSYCHOANALEPTICS	ANTIDEPRESSANTS	ANTIDEPRESSANTS
CLONAZEPAM	NERVOUS SYSTEM	PSYCHOLEPTICS	ANXIOLYTICS	ANXIOLYTICS
CLONIDINE	NERVOUS SYSTEM	PSYCHOANALEPTICS	PSYCHOSTIMULANTS, AGENTS USED FOR ADHD AND NOOTROPICS	OTHER
CLORAZEPIC ACID	NERVOUS SYSTEM	PSYCHOLEPTICS	ANXIOLYTICS	ANXIOLYTICS
CLOZAPINE	NERVOUS SYSTEM	PSYCHOLEPTICS	ANTIPSYCHOTICS	ANTIPSYCHOTICS
CODEINE	NERVOUS SYSTEM	ANALGESICS	OPIOIDS	ANALGESICS
DARIDOREXANT	NERVOUS SYSTEM	PSYCHOLEPTICS	HYPNOTICS AND SEDATIVES	HYPNOTICS
DESIPRAMINE	NERVOUS SYSTEM	PSYCHOANALEPTICS	ANTIDEPRESSANTS	ANTIDEPRESSANTS
DESVENLAFAXINE	NERVOUS SYSTEM	PSYCHOANALEPTICS	ANTIDEPRESSANTS	ANTIDEPRESSANTS
DEUTETRABENAZINE	NERVOUS SYSTEM	OTHER NERVOUS SYSTEM DRUGS	OTHER NERVOUS SYSTEM DRUGS	OTHER
DEXAMFETAMINE	NERVOUS SYSTEM	PSYCHOANALEPTICS	PSYCHOSTIMULANTS, AGENTS USED FOR ADHD AND NOOTROPICS	STIMULANTS
DEXMETHYLPHENIDATE	NERVOUS SYSTEM	PSYCHOANALEPTICS	PSYCHOSTIMULANTS, AGENTS USED FOR ADHD AND NOOTROPICS	STIMULANTS
DEXTROMETHORPHAN;QUINIDINE	NERVOUS SYSTEM	OTHER NERVOUS SYSTEM DRUGS	OTHER NERVOUS SYSTEM DRUGS	OTHER
DEXTROMETHORPHAN/DEXTROMETHORPHAN HYDROBROMIDE;DOXYLAMINE SUCCINATE;EPHEDRINE SULFATE;ETHANOL;PARACETAMOL/DEXTROMETHORPHAN;DOXYLAMINE;EPHEDRINE;ETHANOL;PARACETAMOL/DEXTROMETHORPHAN;DOXYLAMINE;PARACETAMOL/DEXTROMETHORPHAN;GUAIFENESIN;PARACETAMOL;PHENYLEPHRINE/DEXTROMETHORPHAN;GUAIFENESIN;PARACETAMOL;PSEUDOEPHEDRINE/DEXTROMETHORPHAN;PARACETAMOL;PHENYLEPHRINE	NERVOUS SYSTEM	ANALGESICS	OTHER ANALGESICS AND ANTIPYRETICS	ANALGESICS
DIAZEPAM	NERVOUS SYSTEM	PSYCHOLEPTICS	ANXIOLYTICS	ANXIOLYTICS

DICHLORALPHENAZONE;ISOMETHEPTENE;PARA CETAMOL	NERVOUS SYSTEM	ANALGESICS	OTHER ANALGESICS AND ANTIPYRETICS	ANALGESICS
DIMENHYDRINATE	NERVOUS SYSTEM	OTHER NERVOUS SYSTEM DRUGS	ANTIVERTIGO PREPARATIONS	OTHER
DISULFIRAM	NERVOUS SYSTEM	OTHER NERVOUS SYSTEM DRUGS	DRUGS USED IN ADDICTIVE DISORDERS	OTHER
DONEPEZIL	NERVOUS SYSTEM	PSYCHOANALEPTICS	ANTI-DEMENTIA DRUGS	OTHER
DONEPEZIL HYDROCHLORIDE;MEMANTINE HYDROCHLORIDE	NERVOUS SYSTEM	PSYCHOANALEPTICS	ANTI-DEMENTIA DRUGS	OTHER
DOXAZOSIN	NERVOUS SYSTEM	PSYCHOLEPTICS	ANXIOLYTICS	ANXIOLYTICS
DOXEPIN	NERVOUS SYSTEM	PSYCHOLEPTICS	HYPNOTICS AND SEDATIVES	ANTIDEPRESSANTS
DOXYLAMINE	NERVOUS SYSTEM	PSYCHOLEPTICS	HYPNOTICS AND SEDATIVES	HYPNOTICS
DULOXETINE	NERVOUS SYSTEM	PSYCHOANALEPTICS	ANTIDEPRESSANTS	ANTIDEPRESSANTS
ELETRIPTAN	NERVOUS SYSTEM	ANALGESICS	ANTIMIGRAINE PREPARATIONS	ANALGESICS
EPTINEZUMAB	NERVOUS SYSTEM	ANALGESICS	ANTIMIGRAINE PREPARATIONS	ANALGESICS
ERENUMAB	NERVOUS SYSTEM	ANALGESICS	ANTIMIGRAINE PREPARATIONS	ANALGESICS
ESCITALOPRAM	NERVOUS SYSTEM	PSYCHOANALEPTICS	ANTIDEPRESSANTS	ANTIDEPRESSANTS
ESKETAMINE	NERVOUS SYSTEM	PSYCHOANALEPTICS	ANTIDEPRESSANTS	ANTIDEPRESSANTS
ESTAZOLAM	NERVOUS SYSTEM	PSYCHOLEPTICS	HYPNOTICS AND SEDATIVES	HYPNOTICS
ESZOPICLONE	NERVOUS SYSTEM	PSYCHOLEPTICS	HYPNOTICS AND SEDATIVES	HYPNOTICS
FENTANYL	NERVOUS SYSTEM	ANALGESICS	OPIOIDS	ANALGESICS
FLUOXETINE	NERVOUS SYSTEM	PSYCHOANALEPTICS	ANTIDEPRESSANTS	ANTIDEPRESSANTS
FLUPHENAZINE	NERVOUS SYSTEM	PSYCHOLEPTICS	ANTIPSYCHOTICS	ANTIPSYCHOTICS
FLURAZEPAM	NERVOUS SYSTEM	PSYCHOLEPTICS	HYPNOTICS AND SEDATIVES	HYPNOTICS
FLUVOXAMINE	NERVOUS SYSTEM	PSYCHOANALEPTICS	ANTIDEPRESSANTS	ANTIDEPRESSANTS
FREMANEZUMAB VFRM	NERVOUS SYSTEM	ANALGESICS	ANTIMIGRAINE PREPARATIONS	ANALGESICS
GABAPENTIN	NERVOUS SYSTEM	ANTIEPILEPTICS	ANTIEPILEPTICS	ANTIEPILEPTICS
GALCANEZUMAB	NERVOUS SYSTEM	ANALGESICS	ANTIMIGRAINE PREPARATIONS	ANALGESICS
GUANFACINE	NERVOUS SYSTEM	PSYCHOANALEPTICS	PSYCHOSTIMULANTS, AGENTS USED FOR ADHD AND NOOTROPICS	STIMULANTS
HALOPERIDOL	NERVOUS SYSTEM	PSYCHOLEPTICS	ANTIPSYCHOTICS	ANTIPSYCHOTICS
HYDROCODONE	NERVOUS SYSTEM	ANALGESICS	OPIOIDS	ANALGESICS
HYDROCODONE;PARACETAMOL	NERVOUS SYSTEM	ANALGESICS	OPIOIDS	ANALGESICS
HYDROMORPHONE	NERVOUS SYSTEM	ANALGESICS	OPIOIDS	ANALGESICS
HYDROXYZINE	NERVOUS SYSTEM	PSYCHOLEPTICS	ANXIOLYTICS	ANXIOLYTICS
IMIPRAMINE	NERVOUS SYSTEM	PSYCHOANALEPTICS	ANTIDEPRESSANTS	ANTIDEPRESSANTS
ISOCARBOXAZID	NERVOUS SYSTEM	PSYCHOANALEPTICS	ANTIDEPRESSANTS	ANTIDEPRESSANTS

KETAMINE	NERVOUS SYSTEM	PSYCHOANALEPTICS	ANTIDEPRESSANTS	ANTIDEPRESSANTS
LACOSAMIDE	NERVOUS SYSTEM	ANTIEPILEPTICS	ANTIEPILEPTICS	ANTIEPILEPTICS
LAMOTRIGINE	NERVOUS SYSTEM	ANTIEPILEPTICS	ANTIEPILEPTICS	ANTIEPILEPTICS
LASMIDITAN	NERVOUS SYSTEM	ANALGESICS	ANTIMIGRAINE PREPARATIONS	ANALGESICS
LEMBOREXANT	NERVOUS SYSTEM	PSYCHOLEPTICS	HYPNOTICS AND SEDATIVES	HYPNOTICS
LEVETIRACETAM	NERVOUS SYSTEM	ANTIEPILEPTICS	ANTIEPILEPTICS	ANTIEPILEPTICS
LEVOMILNACIPRAN	NERVOUS SYSTEM	PSYCHOANALEPTICS	ANTIDEPRESSANTS	ANTIDEPRESSANTS
LISDEXAMFETAMINE	NERVOUS SYSTEM	PSYCHOANALEPTICS	PSYCHOSTIMULANTS, AGENTS USED FOR ADHD AND NOOTROPICS	STIMULANTS
LITHIUM	NERVOUS SYSTEM	PSYCHOLEPTICS	ANTIPSYCHOTICS	ANTIDEPRESSANTS
LORAZEPAM	NERVOUS SYSTEM	PSYCHOLEPTICS	ANXIOLYTICS	ANXIOLYTICS
LUMATEPERONE	NERVOUS SYSTEM	PSYCHOLEPTICS	ANTIPSYCHOTICS	ANTIPSYCHOTICS
LURASIDONE	NERVOUS SYSTEM	PSYCHOLEPTICS	ANTIPSYCHOTICS	ANTIPSYCHOTICS
MECLOZINE	NERVOUS SYSTEM	OTHER NERVOUS SYSTEM DRUGS	ANTIVERTIGO PREPARATIONS	OTHER
MELATONIN	NERVOUS SYSTEM	PSYCHOLEPTICS	HYPNOTICS AND SEDATIVES	HYPNOTICS
MEMANTINE	NERVOUS SYSTEM	PSYCHOANALEPTICS	ANTI-DEMENTIA DRUGS	OTHER
METHADONE	NERVOUS SYSTEM	ANALGESICS	OPIOIDS	OTHER
METHYLPHENIDATE	NERVOUS SYSTEM	PSYCHOANALEPTICS	PSYCHOSTIMULANTS, AGENTS USED FOR ADHD AND NOOTROPICS	STIMULANTS
MIDAZOLAM	NERVOUS SYSTEM	PSYCHOLEPTICS	HYPNOTICS AND SEDATIVES	HYPNOTICS
MILNACIPRAN	NERVOUS SYSTEM	PSYCHOANALEPTICS	ANTIDEPRESSANTS	ANTIDEPRESSANTS
MIRTAZAPINE	NERVOUS SYSTEM	PSYCHOANALEPTICS	ANTIDEPRESSANTS	ANTIDEPRESSANTS
MODAFINIL	NERVOUS SYSTEM	PSYCHOANALEPTICS	PSYCHOSTIMULANTS, AGENTS USED FOR ADHD AND NOOTROPICS	STIMULANTS
MORPHINE	NERVOUS SYSTEM	ANALGESICS	OPIOIDS	ANALGESICS
NALTREXONE	NERVOUS SYSTEM	OTHER NERVOUS SYSTEM DRUGS	DRUGS USED IN ADDICTIVE DISORDERS	OTHER
NARATRIPTAN	NERVOUS SYSTEM	ANALGESICS	ANTIMIGRAINE PREPARATIONS	ANALGESICS
NEFAZODONE	NERVOUS SYSTEM	PSYCHOANALEPTICS	ANTIDEPRESSANTS	ANTIDEPRESSANTS
NICOTINE POLACRILEX	NERVOUS SYSTEM	OTHER NERVOUS SYSTEM DRUGS	DRUGS USED IN ADDICTIVE DISORDERS	OTHER
NORTRIPTYLINE	NERVOUS SYSTEM	PSYCHOANALEPTICS	ANTIDEPRESSANTS	ANTIDEPRESSANTS
OLANZAPINE	NERVOUS SYSTEM	PSYCHOLEPTICS	ANTIPSYCHOTICS	ANTIPSYCHOTICS
OLANZAPINE;SAMIDORPHAN L-MALATE	NERVOUS SYSTEM	PSYCHOLEPTICS	ANTIPSYCHOTICS	ANTIPSYCHOTICS
OXCARBAZEPINE	NERVOUS SYSTEM	ANTIEPILEPTICS	ANTIEPILEPTICS	ANTIEPILEPTICS
OXITRIPTAN	NERVOUS SYSTEM	PSYCHOANALEPTICS	ANTIDEPRESSANTS	ANTIDEPRESSANTS
OXYCODONE	NERVOUS SYSTEM	ANALGESICS	OPIOIDS	ANALGESICS

OXYCODONE;PARACETAMOL	NERVOUS SYSTEM	ANALGESICS	OPIOIDS	ANALGESICS
PALIPERIDONE	NERVOUS SYSTEM	PSYCHOLEPTICS	ANTIPSYCHOTICS	ANTIPSYCHOTICS
PARACETAMOL	NERVOUS SYSTEM	ANALGESICS	OTHER ANALGESICS AND ANTIPYRETICS	ANALGESICS
PARACETAMOL;PHENYLEPHRINE	NERVOUS SYSTEM	ANALGESICS	OTHER ANALGESICS AND ANTIPYRETICS	ANALGESICS
PAROXETINE	NERVOUS SYSTEM	PSYCHOANALEPTICS	ANTIDEPRESSANTS	ANTIDEPRESSANTS
PERPHENAZINE	NERVOUS SYSTEM	PSYCHOLEPTICS	ANTIPSYCHOTICS	ANTIPSYCHOTICS
PHENELZINE	NERVOUS SYSTEM	PSYCHOANALEPTICS	ANTIDEPRESSANTS	ANTIDEPRESSANTS
PRAMIPEXOLE	NERVOUS SYSTEM	ANTI-PARKINSON DRUGS	DOPAMINERGIC AGENTS	OTHER
PRAZOSIN	NERVOUS SYSTEM	PSYCHOLEPTICS	ANXIOLYTICS	ANXIOLYTICS
PREGABALIN	NERVOUS SYSTEM	PSYCHOLEPTICS	ANXIOLYTICS	ANTIEPILEPTICS
PRIMIDONE	NERVOUS SYSTEM	ANTIEPILEPTICS	ANTIEPILEPTICS	ANTIEPILEPTICS
PROPRANOLOL	NERVOUS SYSTEM	PSYCHOLEPTICS	ANXIOLYTICS	ANXIOLYTICS
PROTRIPTYLINE	NERVOUS SYSTEM	PSYCHOANALEPTICS	ANTIDEPRESSANTS	ANTIDEPRESSANTS
QUETIAPINE	NERVOUS SYSTEM	PSYCHOLEPTICS	ANTIPSYCHOTICS	ANTIPSYCHOTICS
RAMELTEON	NERVOUS SYSTEM	PSYCHOLEPTICS	HYPNOTICS AND SEDATIVES	HYPNOTICS
RIMEGEPANT SULFATE	NERVOUS SYSTEM	ANALGESICS	ANTIMIGRAINE PREPARATIONS	ANALGESICS
RISPERIDONE	NERVOUS SYSTEM	PSYCHOLEPTICS	ANTIPSYCHOTICS	ANTIPSYCHOTICS
RIZATRIPTAN	NERVOUS SYSTEM	ANALGESICS	ANTIMIGRAINE PREPARATIONS	ANALGESICS
ROPINIROLE	NERVOUS SYSTEM	ANTI-PARKINSON DRUGS	DOPAMINERGIC AGENTS	OTHER
SALSALATE	NERVOUS SYSTEM	ANALGESICS	OTHER ANALGESICS AND ANTIPYRETICS	ANALGESICS
SELEGILINE	NERVOUS SYSTEM	PSYCHOANALEPTICS	ANTIDEPRESSANTS	ANTIDEPRESSANTS
SERDEXMETHYLPHENIDATE CHLORIDE	NERVOUS SYSTEM	PSYCHOANALEPTICS	PSYCHOSTIMULANTS, AGENTS USED FOR ADHD AND NOOTROPICS	STIMULANTS
SERTRALINE/SERTRALINE HYDROCHLORIDE	NERVOUS SYSTEM	PSYCHOANALEPTICS	ANTIDEPRESSANTS	ANTIDEPRESSANTS
SOLRIAMFETOL	NERVOUS SYSTEM	PSYCHOANALEPTICS	PSYCHOSTIMULANTS, AGENTS USED FOR ADHD AND NOOTROPICS	STIMULANTS
SUMATRIPTAN	NERVOUS SYSTEM	ANALGESICS	ANTIMIGRAINE PREPARATIONS	ANALGESICS
SUVOREXANT	NERVOUS SYSTEM	PSYCHOLEPTICS	HYPNOTICS AND SEDATIVES	HYPNOTICS
TAPENTADOL	NERVOUS SYSTEM	ANALGESICS	OPIOIDS	ANALGESICS
TEMAZEPAM	NERVOUS SYSTEM	PSYCHOLEPTICS	HYPNOTICS AND SEDATIVES	HYPNOTICS
THIORIDAZINE	NERVOUS SYSTEM	PSYCHOLEPTICS	ANTIPSYCHOTICS	ANTIPSYCHOTICS
TOPIRAMATE	NERVOUS SYSTEM	ANTIEPILEPTICS	ANTIEPILEPTICS	ANTIEPILEPTICS
TRAMADOL	NERVOUS SYSTEM	ANALGESICS	OPIOIDS	ANALGESICS
TRANYLCYPROMINE	NERVOUS SYSTEM	PSYCHOANALEPTICS	ANTIDEPRESSANTS	ANTIDEPRESSANTS
TRAZODONE	NERVOUS SYSTEM	PSYCHOANALEPTICS	ANTIDEPRESSANTS	ANTIDEPRESSANTS

TRIAZOLAM	NERVOUS SYSTEM	PSYCHOLEPTICS	HYPNOTICS AND SEDATIVES	HYPNOTICS
UBROGEPANT	NERVOUS SYSTEM	ANALGESICS	ANTIMIGRAINE PREPARATIONS	ANALGESICS
VALBENAZINE TOSILATE	NERVOUS SYSTEM	OTHER NERVOUS SYSTEM DRUGS	OTHER NERVOUS SYSTEM DRUGS	OTHER
VALPROIC ACID	NERVOUS SYSTEM	ANTIEPILEPTICS	ANTIEPILEPTICS	ANTIEPILEPTICS
VARENICLINE	NERVOUS SYSTEM	OTHER NERVOUS SYSTEM DRUGS	DRUGS USED IN ADDICTIVE DISORDERS	OTHER
VENLAFAXINE	NERVOUS SYSTEM	PSYCHOANALEPTICS	ANTIDEPRESSANTS	ANTIDEPRESSANTS
VERAPAMIL	NERVOUS SYSTEM	ANALGESICS	ANTIMIGRAINE PREPARATIONS	ANALGESICS
VILAZODONE	NERVOUS SYSTEM	PSYCHOANALEPTICS	ANTIDEPRESSANTS	ANTIDEPRESSANTS
VILOXAZINE	NERVOUS SYSTEM	PSYCHOANALEPTICS	ANTIDEPRESSANTS	STIMULANTS
VORTIOXETINE	NERVOUS SYSTEM	PSYCHOANALEPTICS	ANTIDEPRESSANTS	ANTIDEPRESSANTS
ZALEPLON	NERVOUS SYSTEM	PSYCHOLEPTICS	HYPNOTICS AND SEDATIVES	HYPNOTICS
ZIPRASIDONE	NERVOUS SYSTEM	PSYCHOLEPTICS	ANTIPSYCHOTICS	ANTIPSYCHOTICS
ZOLMITRIPTAN	NERVOUS SYSTEM	ANALGESICS	ANTIMIGRAINE PREPARATIONS	ANALGESICS
ZOLPIDEM	NERVOUS SYSTEM	PSYCHOLEPTICS	HYPNOTICS AND SEDATIVES	HYPNOTICS
ZONISAMIDE	NERVOUS SYSTEM	ANTIEPILEPTICS	ANTIEPILEPTICS	ANTIEPILEPTICS
